# Effects of Blood Flow Restriction and Exercise Intensity on Aerobic, Anaerobic, and Muscle Strength Adaptations in Physically Active Collegiate Women

**DOI:** 10.3389/fphys.2019.00810

**Published:** 2019-06-26

**Authors:** Sadegh Amani-Shalamzari, Saeedeh Rajabi, Hamid Rajabi, Daniel E. Gahreman, Carl Paton, Mahdi Bayati, Thomas Rosemann, Pantelis Theodoros Nikolaidis, Beat Knechtle

**Affiliations:** ^1^Department of Exercise Physiology, Faculty of Physical Education and Sport Sciences, Kharazmi University, Tehran, Iran; ^2^College of Health and Human Sciences, Charles Darwin University, Darwin, NT, Australia; ^3^Faculty of Health and Sport Science, Eastern Institute of Technology, Napier, New Zealand; ^4^Department of Exercise Physiology, Sports Medicine Research Center, Sport Sciences Research Institute, Tehran, Iran; ^5^Institute of Primary Care, University of Zurich, Zurich, Switzerland; ^6^Exercise Physiology Laboratory, Nikaia, Greece; ^7^Medbase St. Gallen Am Vadianplatz, St. Gallen, Switzerland

**Keywords:** occlusion, VO_2_max, time to fatigue, running economy, strength

## Abstract

The purpose of this study was to compare the effects of different combinations of blood flow restriction (BFR) pressure and exercise intensity on aerobic, anaerobic, and muscle strength adaptations in physically active collegiate women. Thirty-two women (age 22.8 ± 2.9 years; body mass index 22.3 ± 2.7 kg/m^2^) were randomly assigned into four experimental training groups: (a) increasing BFR pressure with constant exercise intensity (IP-CE), (b) constant partial BFR pressure with increasing exercise intensity (CP_p_-IE), (c) constant complete BFR pressure with increasing exercise intensity (CP_C_-IE), and (d) increasing BFR pressure with increasing exercise intensity (IP-IE). The participants completed 12 training sessions comprised of repeated bouts of 2 min running on a treadmill with BFR interspersed by 1-min recovery without BFR. Participants completed a series of tests to assess muscle strength, aerobic, and anaerobic performances. Muscle strength, anaerobic power, and aerobic parameters including maximum oxygen consumption (VO_2_max), time to fatigue (TTF), velocity at VO_2_max (vVO_2_max), and running economy (RE) improved in all groups (*p* ≤ 0.01). The CP_C_-IE group outscored the other groups in muscle strength, RE, and TTF (*p* < 0.05). In summary, participants with complete occlusion experienced the greatest improvements in muscle strength, aerobic, and anaerobic parameters possibly due to increased oxygen deficiency and higher metabolic stress.

## Introduction

Endurance training improves performance capacity via targeted muscular and cardiovascular adaptations such as an increase in oxidative enzymes, capillary density, glycogen content, and increases in stroke volume and cardiac hypertrophy (Nadel, 1985). Former research suggests that training adaptations maybe further enhanced by the addition of ischemia to working muscles (Sundberg, 1994). Sundberg (1994) applied 50 mmHg restrictive pressure on one leg during exercise for 4 weeks while the contralateral leg was trained without occlusion, and the results indicated a greater peak oxygen uptake and TTF in the ischemic condition. Thus, the application of exogenous hypoxia may be a potent stimulant for physical adaptation.

Blood flow restriction reduces arterial blood flow to working muscles while also occluding venous return. In BFR conditions, active muscles encounter a ischemia state which imposes a greater metabolic stress on working muscles (Tanimoto et al., 2005). The additional metabolic stress of BFR and especially venous occlusion increases muscle cell swelling (Loenneke et al., 2012), activates intra-cellular anabolic pathways (Abe et al., 2005), and recruits fast-twitch fibers (Yasuda et al., 2009), which are thought to be involved in muscular adaptation. The adaptations to exercise with BFR depend on several factors including the pressure of occlusion (i.e., partial or complete), the type of occlusion (i.e., continuous or intermittent), the intensity of exercise (i.e., low, moderate, or high), and the volume of exercise with BFR.

The pressure of occlusion used in previous studies varied from partial (∼160 mmHg) to complete (∼240–300 mmHg) occlusion (Abe et al., 2010a; Park et al., 2010), although it depend on the limb size and the cuff size (Mattocks et al., 2018). To identify an optimum occlusion pressure, Loenneke et al. (2015) studied the effect of different occlusion pressure and training intensity in young males. The results showed greater muscle activation at higher degrees of occlusion (i.e., 50% arterial occlusion). Also, muscle activation was greater at the higher training intensity (i.e., 30% 1RM compared to 20% 1RM). Interestingly, any additional increase in occlusion pressure higher than 50% arterial occlusion did not result in any greater muscle activation, and neither pressure nor training load affected muscle torque development (Loenneke et al., 2015). However, these findings are inconsistent with other research showing a positive effect of higher degrees of occlusion and reporting a sevenfold increase in growth hormone levels in response to a training session with complete occlusion (Pierce et al., 2006) and a fourfold increase in response to partial occlusion compare to non-BFR groups (Reeves et al., 2006).

In addition to occlusion pressure, training intensity is an important factor affecting adaptations in ischemic conditions. Training with BFR is proposed as an effective training paradigm to enhance both aerobic power (Abe et al., 2010a) and muscle strength (Abe et al., 2006; de Oliveira et al., 2016) without the need for training at high intensity. Hence most researchers have studied the effect of low-intensity resistance or aerobic training with BFR. Abe et al. (2010a) compared the effect of 15 min cycling with BFR at 40% VO_2_max with 45 min cycling without BFR at the same intensity for 8 weeks in young men. Their results showed that cycling with BFR (160–210 mmHg) significantly improved VO_2_max, time to exhaustion (TTE), strength, and muscle cross-sectional area (CSA) compared with cycling without BFR. Similarly, Park et al. (2010) reported ∼12% improvements in maximum aerobic capacity and 2.5% increase in anaerobic capacity after 2 weeks of walk training with BFR in collegiate basketball players. Other researchers have also reported an improvement in VO_2_max, onset blood lactate accumulation (OBLA), maximal power output, and muscle strength in BFR group in response to low-intensity aerobic training (Abe et al., 2010a, b; Park et al., 2010; de Oliveira et al., 2016). These adaptations were similar to those seen with high-intensity aerobic interval training without BFR (Laursen and Jenkins, 2002).

A few studies have examined the effects of high-intensity training in combination with BFR. Following 6 (Keramidas et al., 2012) and 4 weeks (Paton et al., 2017) of high-intensity training, there were similar improvements at VO_2_max between the BFR and the non-BFR group, but there were significant improvement at the RE and TTE in BFR group compared to non-BFR group in a former study (Paton et al., 2017). Therefore, it appears that higher intensity training with BFR could also result in additional benefits. However, training at a higher intensity with complete occlusion may be difficult for many individuals because this combination results in higher rate of perceived exertion (RPE) during training (Loenneke et al., 2013; Amani-shalamzari et al., 2019).

Previous studies have investigated the effects of various degrees of BFR or training intensities separately. However, in BFR training, the magnitude of pressure (partial or complete occlusion) and exercise intensity are important variables, which determine physiological adaptations. No previous study has investigated the combination of these variables on physiological and performance measures. It was hypothesized that various combinations of cuff pressure and training intensities result in different aerobic and anaerobic adaptations. Therefore, this study aimed to investigate the effects of various combinations of occlusion pressure, and exercise intensity on performance in physically active collegiate women.

## Materials and Methods

### Experimental Design

This study was a randomized controlled trial and examined the effects of four training protocols with BFR on aerobic and anaerobic adaptations. The protocols were developed using a combination of different occlusion pressure and training intensity. The protocols were chosen based on the most common variations in pressures and exercise intensities used in previous studies. We also took an applied approach and chose to use those methods that were practicable for athletes and coaches to perform during training. The dependent variables in this study included: body composition, aerobic and anaerobic capacity, TTE, vVO_2_max, isometric leg strength, and RE, which were measured before and after the training intervention.

### Participants

Thirty-two active collegiate women met the inclusion criteria and volunteered to participate in this study (Table 1). Eligibility criteria included physically active women aged between 18 and 30 years without orthopedic, neuromuscular disorder, or metabolic and cardiovascular diseases. All experimental procedures were approved by the Ethics committee of Sport Sciences Research Institute of Iran with code IR.SSRI.REC.1397.326 and were conducted in accordance with the Declaration of Helsinki. The researcher explained the risks and benefits of the study to participants and obtained written informed consent prior to initial assessments.

**TABLE 1:** Anthropometric characteristics of participants.

**Group**	**Age (years)**	**Height (m)**	**Body mass (kg)**		**BMI (kg/m^2^)**		**Body fat (%)**	
			**Pre**	**Post**	**Pre**	**Post**	**Pre**	**Post**
IP-CE	22.3 ± 2.4	1.63 ± 0.38	58.7 ± 9.7	56.3 ± 8.8	21.9 ± 3.7	21.4 ± 3.5	23.8 ± 5.6	23.7 ± 2.6
CP_P_-IE	24.3 ± 4.0	1.66 ± 0.55	64.7 ± 7.8	62.8 ± 6.5	23.3 ± 3.2	22.3 ± 2.6	28.9 ± 3.3	26.6 ± 2.7
CP_C_-IE	22.7 ± 2.6	1.66 ± 0.68	61.8 ± 8.8	60.3 ± 7.9	22.5 ± 2.0	22.1 ± 1.6	25.1 ± 4.3	23.1 ± 1.4
IP-IE	21.8 ± 2.2	1.65 ± 4.9	58.2 ± 5.3	57.3 ± 4.8	21.7 ± 1.7	21.1 ± 2.1	25.3 ± 3.4	24.4 ± 2.1

*IP-CE, increasing BFR pressure with constant exercise intensity; CP_*P*_-IE, constant partial BFR pressure with increasing exercise intensity; CP_*C*_-IE, constant complete BFR pressure with increasing exercise intensity; IP-IE, increasing BFR pressure with increasing exercise intensity.*

### Familiarization and Training Programs

**FIGURE 1:**
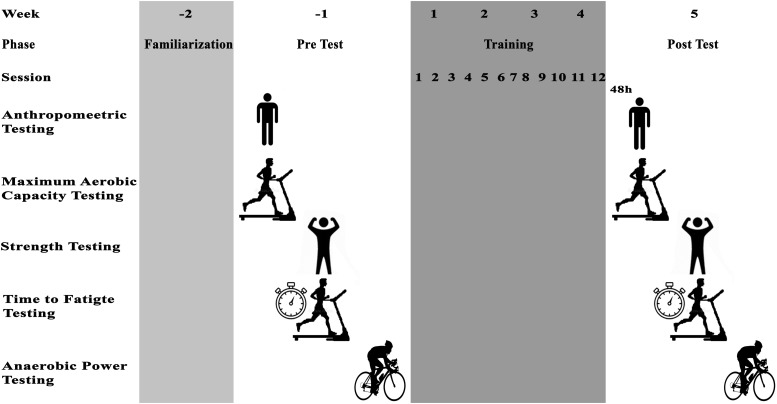
Schematic overview of study timeline.

Participants were familiarized with assessments and training protocols and then the next week anthropometric characteristics, VO_2_max, isometric leg strength, TTE, and anaerobic performance were assessed in three sessions interspersed by 48 h of recovery (Figure 1).

Upon completion of the initial assessment, the participants were divided into four homogeneous groups (*n* = 8) based on aerobic and anaerobic power, then randomly assigned into one of the following four groups: IP-CE, CP_P_-IE, CP_C_-IE, and IP-IE. The intervention period was 4 weeks for all groups and consisted of three sessions per week for 4 weeks (i.e., a total of 12 training sessions). The training protocol for all groups was the same and comprised of bouts of 2 min running on a treadmill with BFR interspersed by 1 min of recovery without BFR. The IP-CE, CP_P_-IE, and CP_C_-IE groups completed 10 sets of training in each session, while the IP-IE group completed 10, 8, 6, and 5 bouts in weeks 1, 2, 3, and 4, respectively. This reduction in training bouts was to account for difficulties in training at high intensity with a complete occlusion. Two 5 × 120 cm pneumatic cuffs (Ghamat Pooyan, Tehran, Iran) were worn by participants on the most proximal portion of both thighs. The cuffs were inflated to the target pressure during the exercise and deflated in the break periods. The final pressure was set to a percentage of arterial occlusion estimated from thigh circumference (Loenneke et al., 2015). The thigh circumference was measured and the partial BFR pressure (∼50% estimated arterial occlusion) and complete occlusion pressure proportional to the thigh circumference were selected. These BFR pressures remained constant in CP_P_-IE and CP_C_-IE groups until the end of the training protocol (Table 2). However, the BFR gradually increased in IP-CE and IP-IE groups. A polar telemetry system (Polar RC3, Polar Electro Oy, Kempele, Finland) was used to measure heart rate during all training sessions. The Borg scale (CR-20) was also used to determine RPE during training (Borg, 1998).

**TABLE 2:** Training protocol for four groups across 4 weeks.

**Groups**		**Week 1**	**Week 2**	**Week 3**	**Week 4**
IP-CE	Cuff pressure (mmHg)	160	190	210	240
	Intensity (%vVO_2_max)	60	60	60	60
CP_P_-IE	Cuff pressure (mmHg)	160	160	160	160
	Intensity (%vVO_2_max)	60	70	80	85
CP_C_-IE	Cuff pressure (mmHg)	240	240	240	240
	Intensity (%vVO_2_max)	60	70	80	85
IP-IE	Cuff pressure (mmHg)	160	190	210	240
	Intensity (%vVO_2_max)	60	70	80	85

*IP-CE, increasing BFR pressure with constant exercise intensity; CP_*P*_-IE, constant partial BFR pressure with increasing exercise intensity; CP_*C*_-IE, constant complete BFR pressure with increasing exercise intensity; IP-IE, increasing BFR pressure with increasing exercise intensity.*

### Test Procedures

Participants attended an Exercise and Sport Science laboratory to complete fitness tests 1 week before and after the training intervention. A schematic view of the testing timeline is presented in Figure 1. Anthropometric measurements included body mass (digital weighing scales, Seca 769, Germany), body height (stadiometer, Seca 213, Germany), and percentage body fat (InBody S10, Biospace Co. Ltd., Seoul, Korea) and were obtained 1 week prior to the intervention period. On the same day, VO_2_max was measured using an incremental running test on a motorized treadmill (Pulsar^®^ 3p, h/p/Cosmos, Nussdorf-Traunstein, Germany). The test started at 10 km h^–1^ for 3 min followed by increments of 1 km h^–1^ every 1 min at 1% inclination until voluntary exhaustion (Milioni et al., 2016). The maximum oxygen uptake and produced carbon dioxide were analyzed breath-by-breath using a calibrated ergospirometer (MetaLyzer3B, CORTEX, Leipzig, Germany). vVO_2_max was defined as the minimum velocity at which VO_2_max was initially achieved (McLaughlin et al., 2010). In addition, a measure of RE was identified using the mean absolute oxygen cost (l min^–1^) measured during the final 2 min of the 10-km/h running stage. TTF was measured 48 h after the VO_2_max-test. After a 5 min warm-up including walking and jogging on a treadmill, participants started the TTF test on a treadmill at a speed corresponding to 60% of their vVO_2_max, and then progressively increased to 100% vVO_2_max over 30–45 s period. At this moment, a stopwatch was used to record the time (the start of vVO_2_max) to exhaustion (Billat et al., 1996). The test was terminated when a participant could not maintain the running speed and reached exhaustion. On the same day, 2 h before TTF test, participants performed an isometric leg muscle strength test using a digital dynamometer (Backmuscle0033, Danesh Salar Iranian, Tehran, Iran) to obtain back and leg strength. Participants stood upright on the base of the dynamometer and adjusted the length of the chain so that the angles of their knees were approximately 110 degrees. With straight arms and without changing the angle of their back, participants pulled the chain steadily with maximum strength. On the following day, participants performed a 30-s Wingate test on a stationary bicycle ergometer (Monark Model 894E, Monark, Vansbro, Sweden) to measure peak power (PP), average power (AP), and minimum power (MP). After cycling for 5 min at 60 revolutions per min (rpm) for warm-up, participants increased the pedaling rate to reach the maximum cadence, and manually dropped the basket that was holding the load equivalent to 0.075 kg kg body mass^–1^. The researchers encouraged participants verbally for the duration of the test.

### Statistical Analysis

Data were analyzed using the statistical package of Social Sciences (SPSS, IBM, v19) and presented in mean ± standard deviation. A one-way analysis of covariance (ANCOVA) was performed to analyze differences between the values of post-test between groups, while the baseline values were considered as co-variants. First, the assumptions of ANCOVA included the normality of data (Shapiro–Wilk test), homogeneity of variance (box plot), and homogeneity of regression slopes was confirmed. To determine differences between groups, when a significant difference was found, Bonferroni *post hoc* tests were performed. Paired sample tests were performed to analyze differences within groups. Effect size (ES) was also calculated to examine the magnitude of differences in the dependent measures within groups, with 0.2 considered as a small ES, 0.5 as a moderate ES, and >0.8 as a large ES (Batterham and Hopkins, 2006). The level of significance was set at *p* ≤ 0.05 for all analysis.

## Results

### Aerobic Parameters

Changes in aerobic and anaerobic fitness, RE, TTF, and effect sizes are presented in Table 3. VO_2_max increased significantly in all groups from pre-to-post intervention (*p* < 0.05). However, the percentage change was different between groups (IP-CE: 9.6 ± 2.0%; CP_P_-IE: 11.2 ± 5.5%; CP_C_-IE: 14.8 ± 4.9%; IP-IE: 8.4 ± 2.4%). Bonferroni *post hoc* analysis revealed a significant difference between CP_C_-IE with IP-IE group (*p* = 0.01).

**TABLE 3:** Adaptations to 4 weeks interval training with BFR.

**Variable**	**Groups**	**Pre**	**Post**	***t*-test *p***	**Cohen’s *d***	**ANCOVA *p***
Peak power (W kg^–1^)	IP-CE	5.8⁢(1.4)	7.3⁢(1.7)	0.001	0.83	0.021^*^
	CP_P_-IE	5.7⁢(1.0)	7.0⁢(1.2)	0.012	0.87	
	CP_C_-IE	5.7⁢(0.8)	7.9⁢(1.2)	0.001	2.05	
	IP-IE	5.9⁢(0.9)	$6.9\,{\left(1.1\right)}^{\#}$	0.020	0.82	
Average power (W kg^–1^)	IP-CE	4.5⁢(0.9)	5.4⁢(0.8)	0.012	0.60	0.030^*^
	CP_P_-IE	4.9⁢(0.9)	$5.4\,{\left(0.9\right)}^{\#}$	0.012	0.52	
	CP_C_-IE	4.6⁢(0.7)	5.9⁢(0.7)	0.001	1.54	
	IP-IE	4.5⁢(0.6)	$5.2\,{\left(0.6\right)}^{\#}$	0.010	0.98	
Minimum power (W kg^–1^)	IP-CE	2.8⁢(0.7)	$2.9\,{\left(0.8\right)}^{\#}$	0.009	0.15	0.007^*^
	CP_P_-IE	3.3⁢(0.8)	$3.6\,{\left(0.8\right)}^{\#}$	0.011	0.22	
	CP_C_-IE	3.1⁢(0.5)	3.7⁢(0.6)	0.001	0.90	
	IP-IE	2.9⁢(0.8)	$3.1\,{\left(0.8\right)}^{\#}$	0.003	0.24	
Leg strength (kg)	IP-CE	82.9⁢(14.6)	$101.8\,{\left(11.8\right)}^{\#}$	0.001	1.00	0.001^*^
	CP_P_-IE	82.6⁢(11.9)	$103.4\,{\left(11.5\right)}^{\#}$	0.001	1.26	
	CP_C_-IE	90.9⁢(9.4)	131.8⁢(7.4)	0.001	2.88	
	IP-IE	87.3⁢(11.1)	$110.1\,{\left(14.9\right)}^{\#}$	0.001	1.53	
VO_2_max (ml kg^–1^ min^–1^)	IP-CE	39.7⁢(1.8)	43.9⁢(1.7)	0.001	1.84	0.024^*^
	CP_P_-IE	39.5⁢(3.7)	44.5⁢(3.5)	0.006	1.10	
	CP_C_-IE	38.3⁢(2.3)	44.9⁢(1.7)	0.001	2.28	
	IP-IE	37.7⁢(3.1)	$41.2\,{\left(3.1\right)}^{\#}$	0.001	0.95	
vVO_2_max (km h^–1^)	IP-CE	12.3⁢(0.5)	14.5⁢(1.0)	0.006	3.27	0.218
	CP_P_-IE	12.2⁢(1.2)	14.0⁢(1.7)	0.028	1.23	
	CP_C_-IE	12.7⁢(0.8)	15.5⁢(0.5)	0.001	2.70	
	IP-IE	12.3⁢(1.0)	14.7⁢(0.5)	0.003	1.79	
Time to fatigue (s)	IP-CE	170.7⁢(15.4)	220.3⁢(28.3)	0.001	2.35	0.042^*^
	CP_P_-IE	182.5⁢(38.1)	$226.8\,{\left(59.1\right)}^{\#}$	0.019	0.85	
	CP_C_-IE	216.0⁢(35.5)	310.5⁢(39.6)	0.001	1.89	
	IP-IE	187.7⁢(26.7)	239.3⁢(30.2)	0.002	1.47	
Running economy (diff: L min^–1^ km h^–1^)	IP-CE	3.6⁢(0.4)	$3.6\,{\left(0.4\right)}^{\#}$	0.090	–0.26	0.024^*^
	CP_P_-IE	3.8⁢(0.7)	3.5⁢(0.5)	0.035	–0.42	
	CP_C_-IE	3.6⁢(0.1)	3.1⁢(0.2)	0.001	–3.92	
	IP-IE	3.8⁢(0.7)	$3.6\,{\left(0.6\right)}^{\#}$	0.046	–0.39	

^#^Significantly different from CP_*C*_-IE group (*P* < 0.05). ^*^Significant difference between groups (*P* < 0.05). IP-CE, increasing BFR pressure with constant exercise intensity; CP_*P*_-IE, constant partial BFR pressure with increasing exercise intensity; CP_*C*_-IE, constant complete BFR pressure with increasing exercise intensity; IP-IE, increasing BFR pressure with increasing exercise intensity; W kg^–1^, Watt per kilogram of body mass; Kg, kilogram; S, Second.

There was a significant difference between the groups in RE (*p* = 0.02). Bonferroni *post hoc* tests revealed a significant difference between the CP_C_-IE group with IP-CE (*p* = 0.04) and IP-IE (*p* = 0.04) groups. The RE decreased significantly from pre-to-post intervention for IP-CE (−5.6 ± 5.2%; CP_P_-IE: −9.6 ± 7.4%; CP_C_-IE: −17.6 ± 6.1%). However, this decrease for IP-IE was not statistically significant (−6.3 ± 6.6%).

In addition, TTF increased significantly in all groups, IP-CE: 22.1 ± 5.4%; CP_P_-IE: 18.2 ± 9.9%; CP_C_-IE: 30.3 ± 7.6%; and IP-IE: 21.3 ± 8.5%. Bonferroni *post hoc* analysis showed a significant difference between CP_C_-IE and CP_P_-IE group (*p* = 0.04).

The vVO_2_max also increased significantly in all groups (IP-CE: 14.6 ± 6.9%; CP_P_-IE: 12.4 ± 9.7%; CP_C_-IE: 18.3 ± 2.9%; and IP-IE: 15.9 ± 6.8%). However, there was no significant difference between groups (*p* = 0.21).

### Anaerobic Parameters

The PP increased significantly in all groups (IP-CE: 21.3 ± 5.5%; CP_P_-IE: 17.5 ± 9.7%; CP_C_-IE: 28.1 ± 4.3%; and IP-IE: 13.5 ± 10.0%). Moreover, there was a significant difference between CP_C_-IE with IP-IE group (*p* = 0.02). In addition, AP showed a significant increase in all groups from pre-training to post-training (IP-CE: 15.1 ± 6.8%; CP_P_-IE: 10.3 ± 6.1%; CP_C_-IE: 22.6 ± 4.9%; and IP-IE: 13.9 ± 8.6%). The difference between CP_C_-IE group with CP_P_-IE and IP-IE groups was also significant (*p* < 0.05).

The MP increased significantly in all groups (IP-CE: 4.9 ± 2.7%; CP_P_-IE: 6.9 ± 3.9%; CP_C_-IE: 16.9 ± 9.2%; and IP-IE: 7.4 ± 3.6%). The results of CP_C_-IE group were significantly different from all other groups (*p* < 0.05).

### Strength

Muscle strength increased significantly in all training conditions (IP-CE: 18.8 ± 7.9%; CP_P_-IE: 20.3 ± 6.3%; CP_C_-IE: 31.0 ± 6.2%; and IP-IE: 20.5 ± 2.7%). The strength of CP_C_-IE group was also significantly different from the other three groups (*p* < 0.05).

### RPE and Heart Rate

**FIGURE 2:**
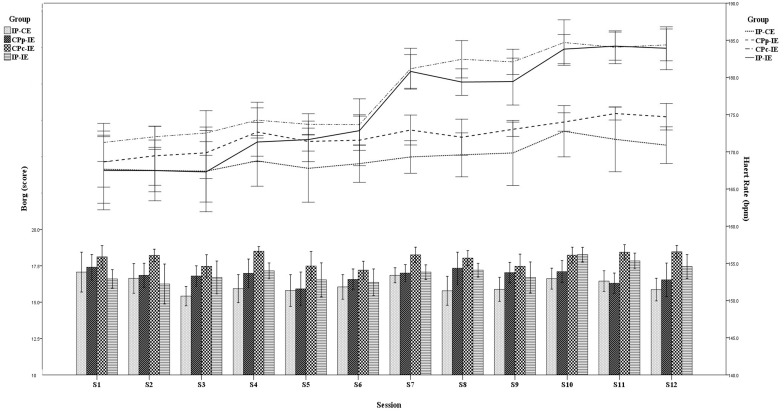
Heart rate responses and the rate of perceived exertion (RPE) for each group in each training session. IP-CE, increasing BFR pressure with constant exercise intensity; CP_P_-IE, constant partial BFR pressure with increasing exercise intensity; CP_C_-IE, constant complete BFR pressure with increasing exercise intensity; IP-IE, increasing BFR pressure with increasing exercise intensity.

Similar to heart rate responses, RPE was greater in the CP_C_-IE group than in the other groups during all training sessions (Figure 2). RPE values for the CP_C_-IE group were “very hard” (18.0 ± 0.4) and for the other groups rated as “hard” (IP-CE: 16.2 ± 0.5; CP_P_-IE: 16.4 ± 0.4; IP-IE: 16.8 ± 0.6). In most training sessions, the mean heart rate was higher in the CP_C_-IE group than in the other groups. During the last week, mean heart rate in CP_C_-IE and IP-IE was the same but higher than in the other two groups (Figure 2).

## Discussion

Previous researchers have examined the efficacy of aerobic training with BFR, and because of methodological difference that included various combinations of exercise loads and occlusion pressures, reported inconsistent and contradictory findings (Sundberg, 1994; Abe et al., 2006; Park et al., 2010). This study attempted to identify an optimal combination of occlusion and exercise intensity to maximize aerobic and anaerobic adaptations. The results showed greater improvements in strength, aerobic, and anaerobic parameters in response to complete occlusion and progressive training.

The improvement in VO_2_max and aerobic parameters such as vVO_2_max and TTF in this study is in agreement with those of previous studies that investigated the effect of low-intensity aerobic exercise in combination with BFR (Abe et al., 2010a, b; Park et al., 2010). Significant improvements in aerobic capacity have been reported in response to 4 weeks of training with 50 mmHg external pressure (Sundberg, 1994), 2 weeks training with 200 mmHg (Park et al., 2010), 4 weeks training with 140–200 mmHg (de Oliveira et al., 2016). These results suggest an interrelationship between the training duration and the degree of BFR, in which training programs with lower training durations could be effective if a higher degree of BFR is imposed and vice versa.

Maximum oxygen consumption is identified by cardiac output and arteriovenous oxygen difference. Hemodynamic parameters such as stroke volume, heart rate, and cardiac output are possible mechanisms that could have contributed to the increased aerobic capacity in this study. Although these factors were not measured in this study, Park et al. (2010) attributed the improvements in aerobic power in response to walking with BFR to hemodynamic parameters. Furthermore, the increased fluid shear stress caused by ischemia and reperfusion between repetitions in this study could have been a strong stimulator for up-regulation of expression of angiogenesis-related factors (Hudlicka and Brown, 2009), which has been shown to improve aerobic power (Hudlicka and Brown, 2009).

Running economy is related to oxygen consumption in running at a specific velocity. The results of this study showed that RE can significantly improve in response to progressive exercise intensity. Thus, exercise intensity appears to be a key factor in improving RE. Chen et al. (2009) compared RE in response to training at 70, 80, and 90% VO_2_max and reported a greater improvement in response to training at a higher intensity (the lactate threshold) compared to low intensity (Chen et al., 2009). Also, an improved RE in response to high-intensity training (80% peak running velocity) and BFR was reported by Paton et al. (2017). Researchers attributed this improvement to intra-muscle adaptations such as the activity of oxidative enzymes and capillary density of involved muscles (Esbjornsson et al., 1993; Paton et al., 2017). In this study, there were significant differences between the RE in CP_C_-IE with IP-CE and IP-IE groups. It seems that in addition to exercise intensity, training volume could also affect the magnitude of change in RE. The training volume of the IP-IE group was reduced over time (from 10 repetitions in the first week to 5 repetitions in the last week). Therefore, to improve the RE, training volume may also need to be considered.

In addition to improvement in aerobic parameters, significant increases with large ES were observed in TTF in all groups. An interesting finding was the larger percentage of change from pre-intervention to post-intervention in groups that experience complete occlusion either from the beginning or toward the end of the training intervention. This finding may suggest that greater cuff pressure may result in greater improvements.

Time to fatigue was defined as the duration of running from vVO_2_max to exhaustion (Messonnier et al., 2002). The energy required for this period is provided by both aerobic and anaerobic energy system (Bertuzzi et al., 2012), and the performance during this time relies on anaerobic capacity and lactate clearance capabilities (Messonnier et al., 2002). It is likely that participants of this study have developed greater anaerobic capacity and lactate clearance ability, and these adaptations may have contributed to TTF improvement. Although these variables were not measured in this study, previous studies have demonstrated that training with BFR results in a significant increase in the rate of lactate clearance during incremental exercise (de Oliveira et al., 2016). In addition, it has been reported that training with BFR is associated with increased recruitment of type 2 muscle fibers and a corresponding increase in lactate production (Takano et al., 2005). Thus, increased lactate tolerance with BFR may be associated with a TTF increase. Also, it is possible that muscle buffering capacity have increased in all groups, especially in groups that cuff pressure either was at a maximum or was increasing. In these groups, accumulation of metabolic by-products was associated with more buffering capacity.

The Wingate anaerobic test is considered as the gold standard for assessment of anaerobic power. In this study, significant increases in anaerobic parameters (peak, average, and minimum power) were observed in all groups. This observed improvements in anaerobic parameters in our study were similar to those reported in response to resistance training with BFR (Sundberg, 1994; Burgomaster et al., 2003), and aerobic training with BFR (Park et al., 2010). Based on the results of this study, it seems there was a relationship between the magnitude of BFR and improvements in anaerobic power, so that the least improvement in anaerobic parameters was seen in the CP_P_-IE group where the cuff pressure remained low throughout the intervention period. The pressure of cuff in the other three groups reached the complete occlusion during the intervention, and the differences between these three groups were significantly higher than of those observed in CP_P_-IE group. The difference in PP between CP_C_-IE group with IP-IE group could be attributed to the lower exercise volume in the IP-IE group; although the higher cuff pressure in this group was a plausible reason for the observed non-significant difference with the other two groups. The metabolic stress with BFR training plays an important role in initiating muscle adaptations (Suga et al., 2012; Teixeira et al., 2018). Dependence on anaerobic metabolism and disturbed oxygen delivery during BFR training could result in increased muscle glycogen stores (Sundberg, 1994; Burgomaster et al., 2003) which would improve anaerobic power especially in CP_C_-IE group that trained with the highest metabolic stress.

An important finding of this study was a significant increase in muscle strength in all groups that was reinforced by large effect sizes. Furthermore, CP_C_-IE group showed a significantly larger gain in muscle strength compared to other three groups. Few researches reported an increased muscle strength with short-term low-intensity training (de Oliveira et al., 2016) and walking (Abe et al., 2006, 2010b) with BFR. The mechanisms of strength gain in response to training with BFR are not fully understood. Abe et al. (2006) observed an increase in CSA of thigh muscles in response to 3 weeks of twice-daily KAATSU-walking in young men. A significant increase in muscle strength in all groups in this study could possibly be due to an increase in CSA. However, a limitation of this study was the lack of information about structural changes of the involved muscles.

Another possibility for the observed increase in muscle strength could be the recruitment of fast twitch muscle fibers. Previous researchers have highlighted the effect of oxygen deficiency on the recruitment of fast twitch motor units (type II muscle fibers) to generate force and strength adaptations (Moritani et al., 1992; Abe et al., 2010b; de Oliveira et al., 2016). It seems that BFR which is associated with the release of metabolic by-products could recruit more fast-twitch motor units to generate force, and thus induces a favorable milieu for improving muscle strength and anaerobic power (Moore et al., 2004).

We acknowledge several limitations with this study. First, instead of the gold standard method, such as Doppler, we estimated arterial occlusion from the thigh circumference which is a method based on findings of previous research (Loenneke et al., 2015). Second, the sample size was small because few female students from a large statistical community volunteered to participate in the study and met the inclusion criteria.

## Conclusion

In conclusion, this study showed that training intensity and magnitude of BFR might alter aerobic, anaerobic, and muscular performance in physically active women. Our findings demonstrated improvements in all aerobic and anaerobic variables in all groups with a trend for greater gains in strength, aerobic, and anaerobic parameters in response to progressively periodized intensity and complete occlusion.

## Ethics Statement

All procedures performed in studies involving human participants were in accordance with the ethical standards of Sport Sciences Research Institute of Iran with code IR.SSRI.REC.1397.326 and with the 1964 Helsinki declaration and its later amendments or comparable ethical standards. This article does not contain any studies with animals performed by any of the authors.

## Author Contributions

SA-S, HR, and CP conceived the study. SR and SA-S conducted the experiments. SA-S, DG, CP, and MB analyzed the study. SA-S, HR, DG, CP, MB, TR, PN, and BK interpreted the data for the study. All authors made substantial contributions to the design of the work, drafted the work or revised it critically for important intellectual content, provided final approval of the version to be published, and agreed to be accountable for all aspects of the work in ensuring that questions related to the accuracy or integrity of any part of the work are appropriately investigated and resolved.

## Conflict of Interest Statement

The authors declare that the research was conducted in the absence of any commercial or financial relationships that could be construed as a potential conflict of interest.

## Abbreviations

ANCOVA: analysis of covariance
AP: average power
BFR: blood flow restriction
CP_C_-IE: constant complete BFR pressure with increasing exercise intensity
CP_P_-IE: constant partial BFR pressure with increasing exercise intensity
CSA: cross-sectional area
ES: effect size
IP-CE: increasing BFR pressure with constant exercise intensity
IP-IE: increasing BFR pressure with increasing exercise intensity
MP: minimum power
OBLA: onset blood lactate accumulation
PP: peak power
RE: running economy
RPE: rate perceived exertion
TTE: time to exhaustion
TTF: time to fatigue
VO_2_max: maximum oxygen consumption
vVO_2_max: velocity at VO_2_max.
